# Integration of a Planar Thick-Film CuNi–Cu-Based Thermoelectric Microgenerator with an RFID Tag

**DOI:** 10.3390/s26185842

**Published:** 2026-09-15

**Authors:** Szymon Wójcik, Piotr Markowski, Krzysztof Stojek, Patryk Pyt, Andrzej Dziedzic

**Affiliations:** 1 Faculty of Electronics, Photonics and Microsystems, Wrocław University of Science and Technology, Wybrzeże Wyspiańskiego 27, 50-370 Wrocław, Poland; piotr.markowski@pwr.edu.pl (P.M.); krzysztof.stojek@pwr.edu.pl (K.S.); andrzej.dziedzic@pwr.edu.pl (A.D.); 2 Department of Electronic and Telecommunications Systems, Rzeszów University of Technology, Aleja Powstańców Warszawy 12, 35-029 Rzeszów, Poland; p.pyt@prz.edu.pl

**Keywords:** energy harvesting, thermoelectricity, thick-film, thermocouple, thermopile, thermoelectric microgenerator, RFID tag

## Abstract

This paper presents the integration of a planar thick-film thermoelectric microgenerator based on CuNi–Cu materials with an HF RFID tag STM M24LR64E-R. The developed microgenerator consisted of 20 thermocouples connected in series and was characterized by a total internal resistance of 7.11 Ω. To match the energy parameters of both systems, a low-power LTC3108 DC–DC converter was used, allowing the increase in the voltage generated by the microgenerator from 80 mV to 2.35 V. The output power obtained from the converter was 29.3 µW, which proved sufficient to power the RFID tag. In idle mode, the power consumption of the tag was approximately 17 µW, while during the communication process it increased to 42 µW for approximately 1 ms. Subsequently, for about 25 ms, the energy demand of the system remained below the power delivered by the converter. The energy balance analysis performed demonstrated the possibility of correctly powering the tag throughout the entire communication cycle. The results obtained indicate that the use of a thermoelectric microgenerator as a power source for an RFID tag may contribute to reducing the system response time by eliminating the need for wake-up procedures, as well as potentially increasing the effective communication range.

## 1. Introduction

Radio Frequency Identification (RFID) systems are currently widely used across numerous areas of industry and technology, including logistics and supply chain management, automatic product identification, contactless payment systems, industrial asset tracking, access control, textronic solutions, medicine, and military applications [1,2,3,4,5,6,7,8]. The dynamic development of RFID technology is associated with increasing requirements regarding operational reliability, device miniaturization, and the ability to ensure long-term autonomous operation without the need for replaceable power sources [1].

Although most RFID tags operate in a passive mode, an increasing number of advanced applications require an extension of system functionality through improved communication range, integration of additional sensors, implementation of environmental parameter monitoring, or periodic data logging. These functionalities lead to increased energy demand, which in turn drives the development of energy harvesting technologies. Such technologies enable the reduction or complete elimination of batteries and the need for periodic recharging, thereby enhancing the autonomy of RFID systems [9].

One of the promising energy harvesting methods is the utilization of the thermoelectric effect, which enables the direct conversion of thermal energy into electrical energy. Thermoelectric microgenerators (TEGs) may serve as an alternative power source for ultra-low-power electronic systems, including modern RFID tags [9]. Thick-film technologies represent one of the viable approaches due to their relatively simple fabrication process, low production cost, scalability, and compatibility with printed electronics technologies, making them highly suitable for integration with various electronic systems.

Planar thick-film thermoelectric microgenerators (μTEGs) based on CuNi–Cu thermocouples constitute an interesting alternative to conventional thermoelectric solutions. The use of these materials enables the development of low-cost thermoelectric structures that can be directly integrated with RFID circuits and other printed electronic systems. The planar architecture of such microgenerators facilitates their implementation in compact and autonomous identification and monitoring systems.

This paper presents issues related to the integration of a planar thick-film thermoelectric microgenerator based on CuNi–Cu thermocouples with an RFID tag. The energy requirements of the RFID system, operating conditions of the thermoelectric microgenerator, and the feasibility of practical integration of both components are analyzed. The study also presents experimental results and analyses aimed at evaluating the potential of thick-film thermoelectric microgenerators as a supplementary power source for autonomous RFID systems.

## 2. Integration Requirements

This section presents an analysis of the energy and technological aspects that determine the feasibility of integrating a planar thermoelectric microgenerator with an RFID tag. The limitations arising from the parameters of the employed components are taken into account, including the characteristics of the boost converter and the requirements imposed by the operating mode of the tag. In addition, issues related to technological compatibility and the possibility of effective structural integration of the microgenerator with the RFID system are examined in the context of autonomous identification applications.

### 2.1. RFID Tag

The electrical requirements for powering RFID tags using a planar thermoelectric microgenerator were determined based on a simulation-oriented study [10]. The available literature indicates that the minimum voltage required for proper operation of RFID circuits lies in the range of 1 V to 1.5 V [11,12,13]. It is worth noting that, due to the widespread use of power sources with a nominal voltage of 1.5 V, many low-voltage electronic devices are designed to operate under supply conditions corresponding to this level.

At the same time, it should be emphasized that the key parameter determining the correct operation of RFID systems is not voltage itself, but available power. According to the literature data, the minimum power required for RFID tag operation is approximately 8–10 µW, which enables basic functions such as identifier (ID) readout [2]. In optimized solutions, operation at even lower power consumption levels, down to 3.15 µW, has been reported [9]. Nevertheless, in practical applications, the typical operating power range of RFID tags is assumed to lie between 8 µW and 1 mW [2,14,15].

Based on the conducted analysis, a commercial STM M24LR64E-R device was selected for use, intended for low-power applications. It is a dynamic RFID/NFC tag operating at 13.56 MHz. The device integrates a 64-kbit EEPROM memory and supports the ISO 15693 standard. Additionally, it is equipped with an I^2^C interface, enabling integration with both wireless and wired systems [16].

The device can operate in either passive or semi-passive mode. In passive mode, the energy required for operation and communication is harvested exclusively from the electromagnetic field generated by the RFID reader. In a semi-passive configuration, an external power source can be used to supply part of the circuitry, while radio communication is still powered by energy harvested from the reader’s field [16].

According to the documentation, proper operation of the device requires a stable supply voltage in the range of 1.8–5.5 V applied between the V_CC_ and V_SS_ pins. The device includes an internal voltage regulator that ensures correct operating conditions when powered via the V_CC_ pin and simultaneously prevents voltage originating from the internal RF rectifier from appearing at this pin during exposure to the RFID reader’s electromagnetic field [16].

### 2.2. μTEG

A planar thick-film thermoelectric microgenerator based on CuNi–Cu thermocouples was used to implement the integration [17]. In planar CuNi–Cu structures, the generated voltage, given a known Seebeck coefficient, depends primarily on the number of thermocouples employed and the temperature difference between the hot and cold junctions. Achieving an output voltage in the range of 1.8–5.5 V, required for proper operation of the STM M24LR64E-R tag, is in practice difficult while maintaining the compact dimensions of a thick-film structure.

For this reason, an LTC3108 boost converter was used. It is an ultra-low voltage step-up converter designed for energy harvesting systems, capable of startup at input voltages as low as approximately 20 mV, particularly when used with sources of low internal resistance in the range of 1–10 Ω [18,19]. The primary objective was to connect the microgenerator to the input of the LTC3108 converter, which boosts the input voltage, and subsequently connect the converter output to the STM M24LR64E-R RFID tag. This configuration enables continuous power supply to the tag’s DC circuitry, allowing it to remain continuously operational. The main objective of this study was to verify whether the power generated by the microgenerator is sufficient to maintain the RFID tag in a continuously powered state.

Based on the parameters of the converter’s datasheet and the constraints imposed by thick-film technology, the optimal microgenerator structure was defined. The designed device consists of 20 CuNi–Cu thermocouples and exhibits a theoretical internal resistance of $R_{TEG}=$ 8.43 Ω.

A single thermocouple was composed of Cu and CuNi conductive tracks, with the Cu track having a width of 0.4 mm and the CuNi track a width of 1.2 mm. The spacing between the Cu and CuNi tracks was maintained at 0.4 mm, while the overall length of the thermocouple was 8 mm. The thicknesses of the Cu and CuNi layers were 15 µm and 30 µm, respectively. For the designed geometry, the average electrical resistance of a single thermocouple equals 421.5 mΩ. Assuming a Seebeck coefficient of 42.86 µV/K for a single thermocouple, the input voltage level required for proper operation of the converter can be achieved at a temperature difference of approximately 50 K between the microgenerator junctions.

**Figure 1:**
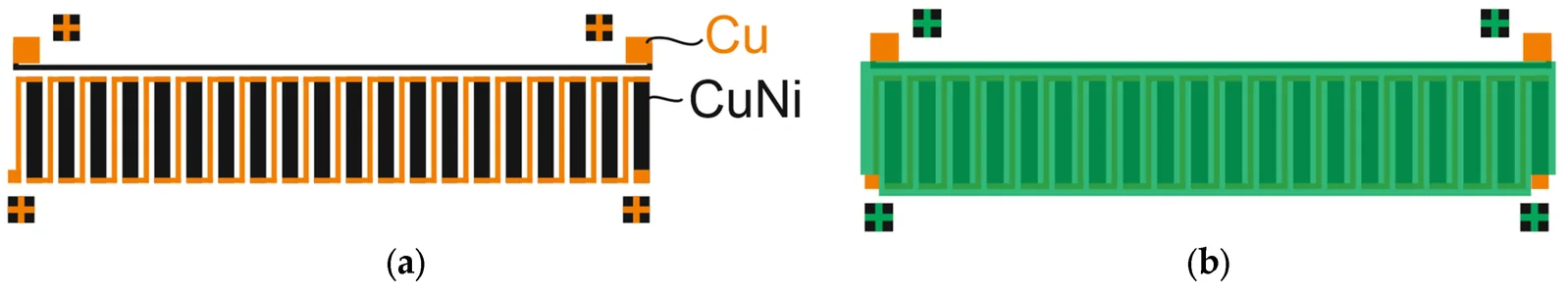
CuNi–Cu microgenerator design: (**a**) structure before glazing; (**b**) structure after glazing.

To protect the copper layers against oxidation, an additional glazing process in a nitrogen atmosphere was introduced using Heraeus IP7098 glass. The structure layout is shown in Figure 1. A heating resistor was also made in the upper part of the design, allowing controlled heating of the hot junction of the microgenerator using a laboratory power supply, providing convenient test conditions. It is worth noting that the same set of materials was used to make the thermoelectric microgenerator and the heating resistor.

## 3. Analysis of Integrated μTEG–RFID Performance

**Figure 2:**
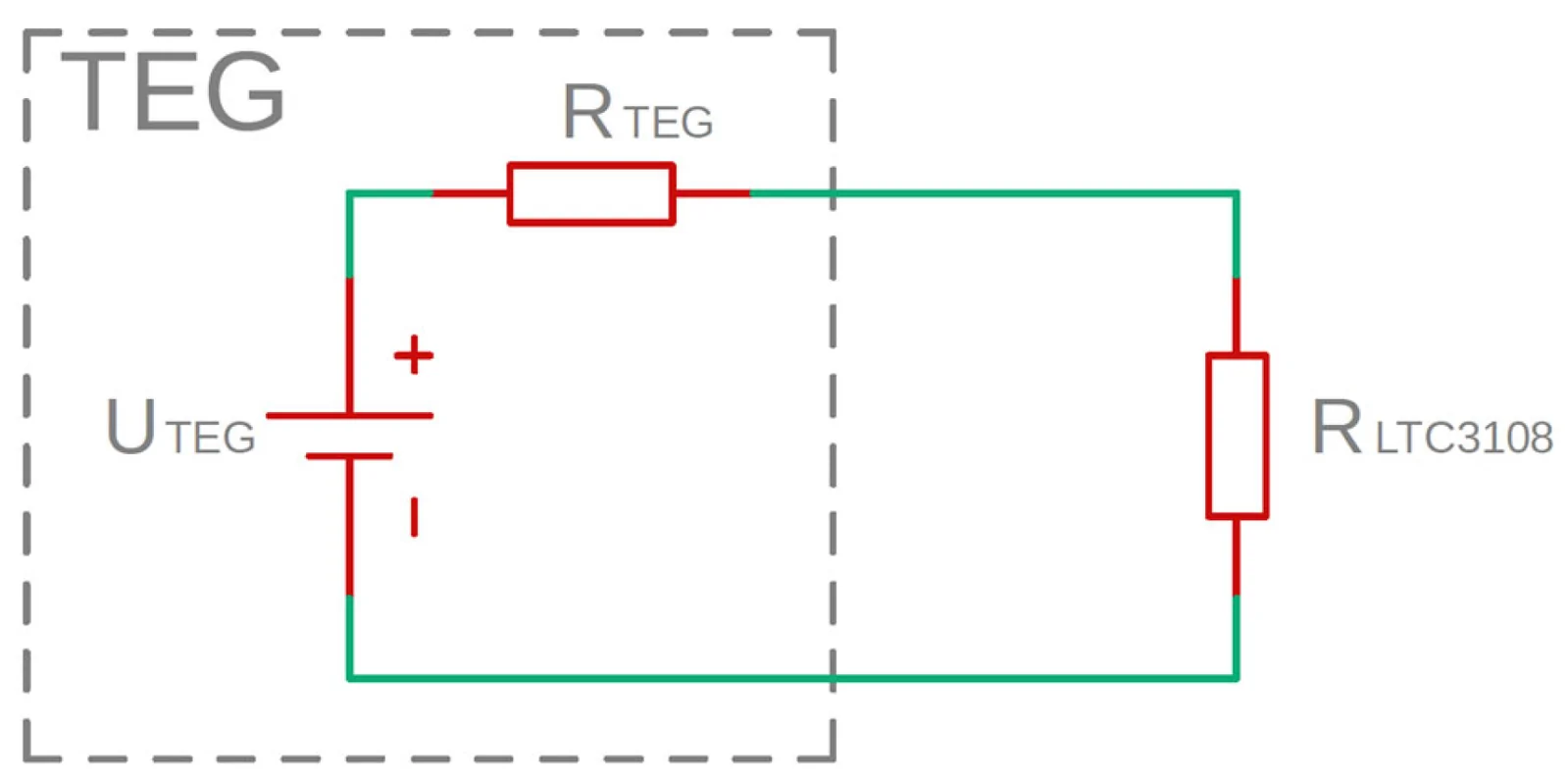
Equivalent model of the microgenerator with the LTC3108 converter input.

For the purpose of the integration analysis, a simplified equivalent model (Figure 2) was developed to estimate the output parameters of the CuNi–Cu μTEG. The model corresponds to a structure with a theoretical internal resistance of $R_{TEG}=$ 8.43 Ω, consisting of 20 thermocouples characterized by a Seebeck coefficient of 42.86 µV/K per thermocouple. The simplified equivalent circuit allowed an approximate analysis of the voltage and power generated by the device under specified temperature difference conditions.

For the calculations, it was assumed that the microgenerator generates an output voltage of 80 mV. Assuming resistive matching, $R_{LTC}\approx R_{TEG}$, the current value was determined according to:

(1) $$I=\frac{U_{OC}}{R_{TEG}+R_{LTC}}=\;\frac{0.08}{16.86}=4.745\;mA$$

where I—current flowing in the circuit [A]; $U_{OC}$—open-circuit voltage of the TEG [V]; $R_{TEG}$—internal resistance of the TEG [Ω]; $R_{LTC}$—input resistance of the LTC3108 converter [Ω].

The obtained result indicates that, under ideal resistive matching conditions, a current of 4.745 mA flows at the input of the LTC3108 converter. In this case, the converter input voltage can be determined according to:

(2) $$U_{LTC}=U_{OC}-I\cdot R_{TEG}=80\;mV-4.745\;mA\;\cdot 8.43\;\Omega =40\;mV$$

where $U_{LTC}$—input voltage of the LTC3108 converter [V].

By taking into account the equivalent source model (Figure 2), it is possible to determine the power delivered to the input of the converter according to:

(3) $$P_{in}=I^{2}\cdot R_{LTC}={\left(\frac{U_{OC}}{R_{TEG}+R_{LTC}}\right)}^{2}\cdot R_{LTC}$$

(4) $$P_{in}={\left(\frac{U_{OC}}{2R}\right)}^{2}\cdot R=\frac{U_{OC}^{2}}{4R}=189.798\;µW$$

where $P_{in}$—input power to the LTC3108 converter [W].

Assuming that $\frac{R_{LTC}}{R_{TEG}}=k$, it is easy to show that the power available at the input of the LTC3108 compared to the power available with resistive matching is described by the relationship (5). The parameter k directly quantifies the degree of resistance mismatch between the converter and the thermoelectric microgenerator. Under ideal matching of the two resistances, the harvested power reaches its maximum value, and the power ratio is equal to unity. It is worth noting that a resistance mismatch of 20% results in only an approximately 1% change in the power ratio. This effect can be observed in Table 2 by comparing Structures 5 and 8. Therefore, the influence of resistance mismatch on the available input power is relatively small.

(5) $$\frac{P_{in}\left(k\cdot R_{TEG}\right)}{P_{in}\left(R_{TEG}\right)}=\frac{4\cdot k}{{\left(k+1\right)}^{2}}$$

According to the literature data, the efficiency of the LTC3108 converter at very low input voltages is typically around 15% [19], which leads to Equation (6).

(6) $$P_{out}=\eta \cdot P_{in}=0.15\cdot 189.798=28.47\;µW$$

where $P_{out}$—output power of the LTC3108 converter [W]; η—efficiency of the LTC3108 converter system [-].

**Figure 3:**
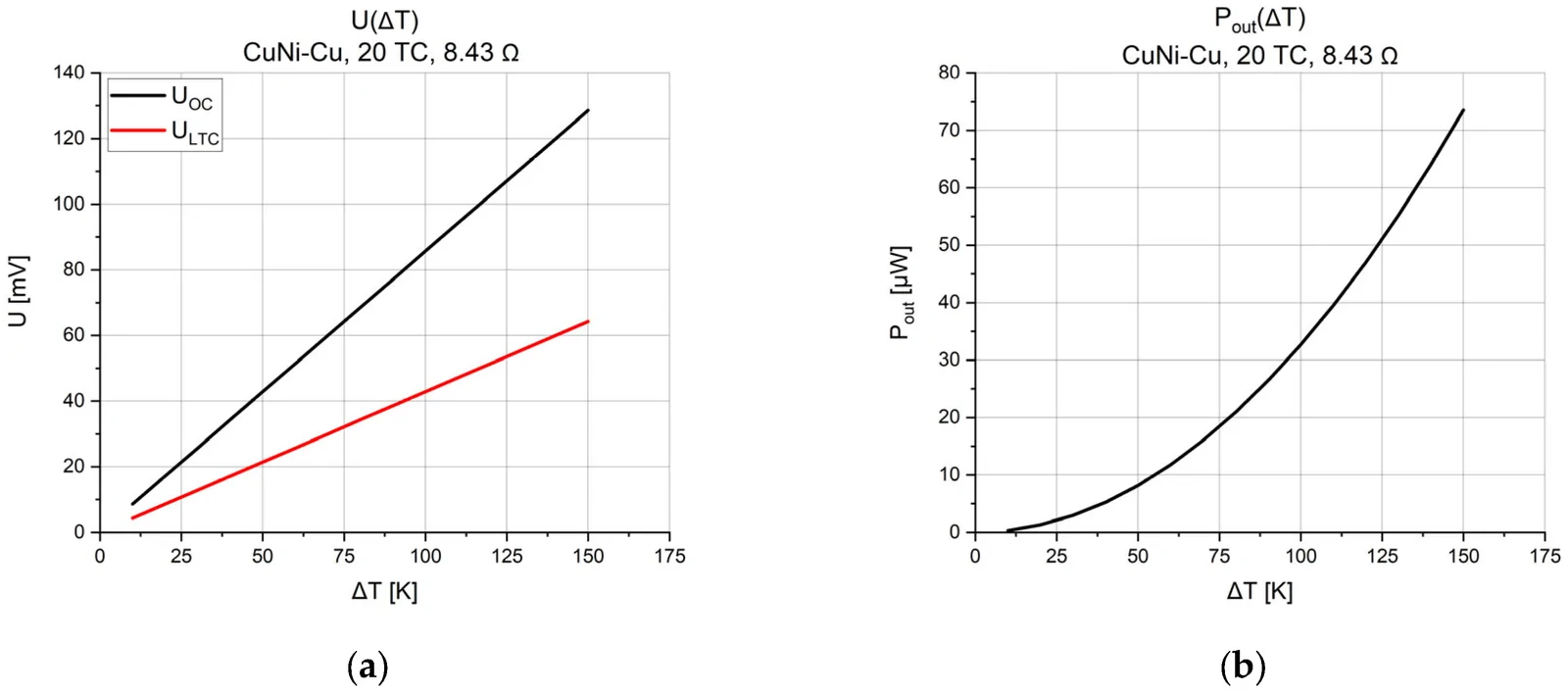
Electrical parameters of the μTEG–LTC3108 integrated system: (**a**) output voltage of the μTEG and input voltage of the converter; (**b**) output power of the LTC3108 converter.

Based on the performed calculations, the theoretical parameters of the simulated structure were determined, as presented in Figure 3.

## 4. Fabrication of CuNi–Cu Thermoelectric Microgenerator

Based on the developed design, appropriate technological masks were prepared, followed by the fabrication of three screens dedicated to the screen-printing process. The screen-printing process was carried out using a UniPrint PMGo3V printer. Each film was dried in a Binder FED 56 dryer, while the final firing process was performed in a BTU QA 41-6-54 belt furnace. The materials used are summarized in Table 1.

**Figure 4:**
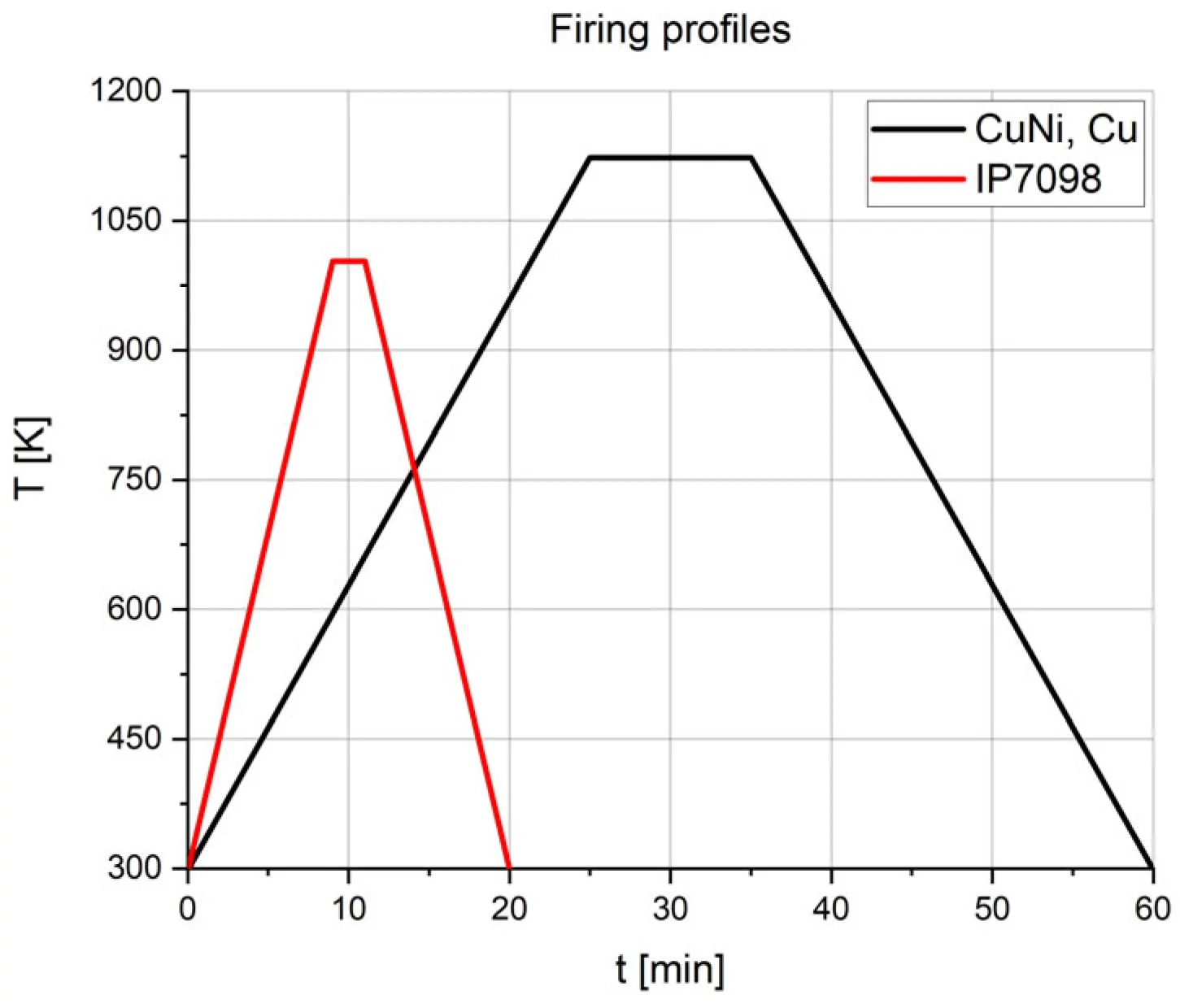
Paste firing profiles.

All firing processes were carried out in a protective nitrogen atmosphere. The structures were fabricated on an Al_2_O_3_ substrate with dimensions of 50 × 30 mm. In the first stage, the CuNi paste was screen-printed twice in order to reduce the resistance of the layer, followed by the application of the Cu paste. After each deposition step, the layers were dried for 10 min. Subsequently, co-firing of the layers was performed according to the appropriate temperature profile shown in Figure 4.

After the firing process, a glazing step was carried out to protect the conductive films against oxidation under elevated operating temperatures. The glass film was applied twice, with a drying stage performed after each deposition. Finally, the glaze was fired according to the temperature profile shown in Figure 4.

**Figure 5:**
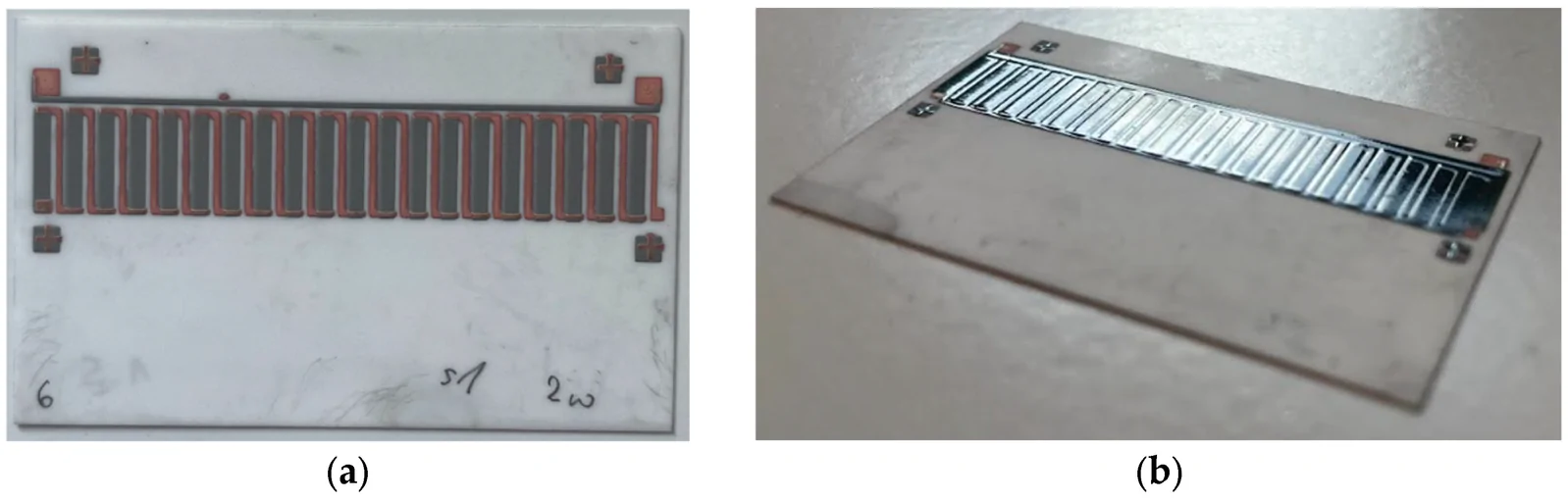
CuNi–Cu structures: (**a**) before glazing; (**b**) after glazing.

The fabricated structures are presented in Figure 5, while their properties are summarized in Table 2.

**Table 2:** Parameters of the fabricated structures.

Parameter	1	2	3	4	5	6	7	8
*R_TEG_* [Ω]	12.94	12.33	9.59	9.36	8.54	8.32	8.19	7.11
*k* = *R_LTC_/R_TEG_* [-]	0.65	0.68	0.88	0.90	0.99	1.01	1.03	1.19
*P_in_* (*R_LTC_*)/*P_in_* (*R_TEG_*)	0.955	0.964	0.996	0.997	0.999	0.999	0.999	0.992

Based on the obtained structures, the variant with the lowest resistance was selected, since its reduction leads to an increase in the output power generated by the microgenerator. The structure with a resistance of 7.11 Ω, at an open-circuit voltage $U_{OC}$ = 80 mV, is expected to generate an input power to the converter of $P_{in}$ = 225 µW, while the output power of the converter is $P_{out}$ = 33.76 µW.

## 5. Integration

**Figure 6:**
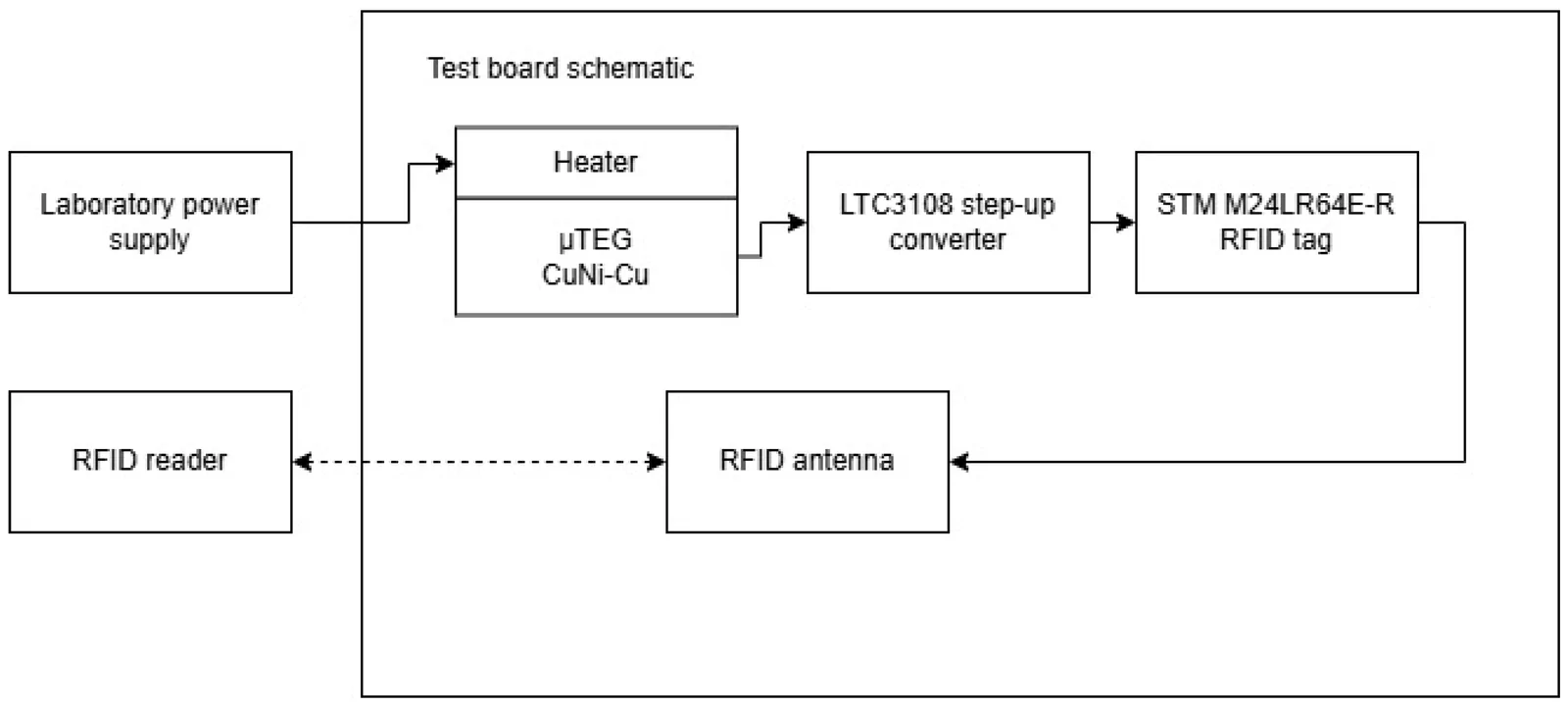
Test board schematic.

After selecting all components, a test board was designed to arrange the system elements and ensure full integration of the system for further investigations. The block diagram of the developed solution is shown in Figure 6.

**Figure 7:**
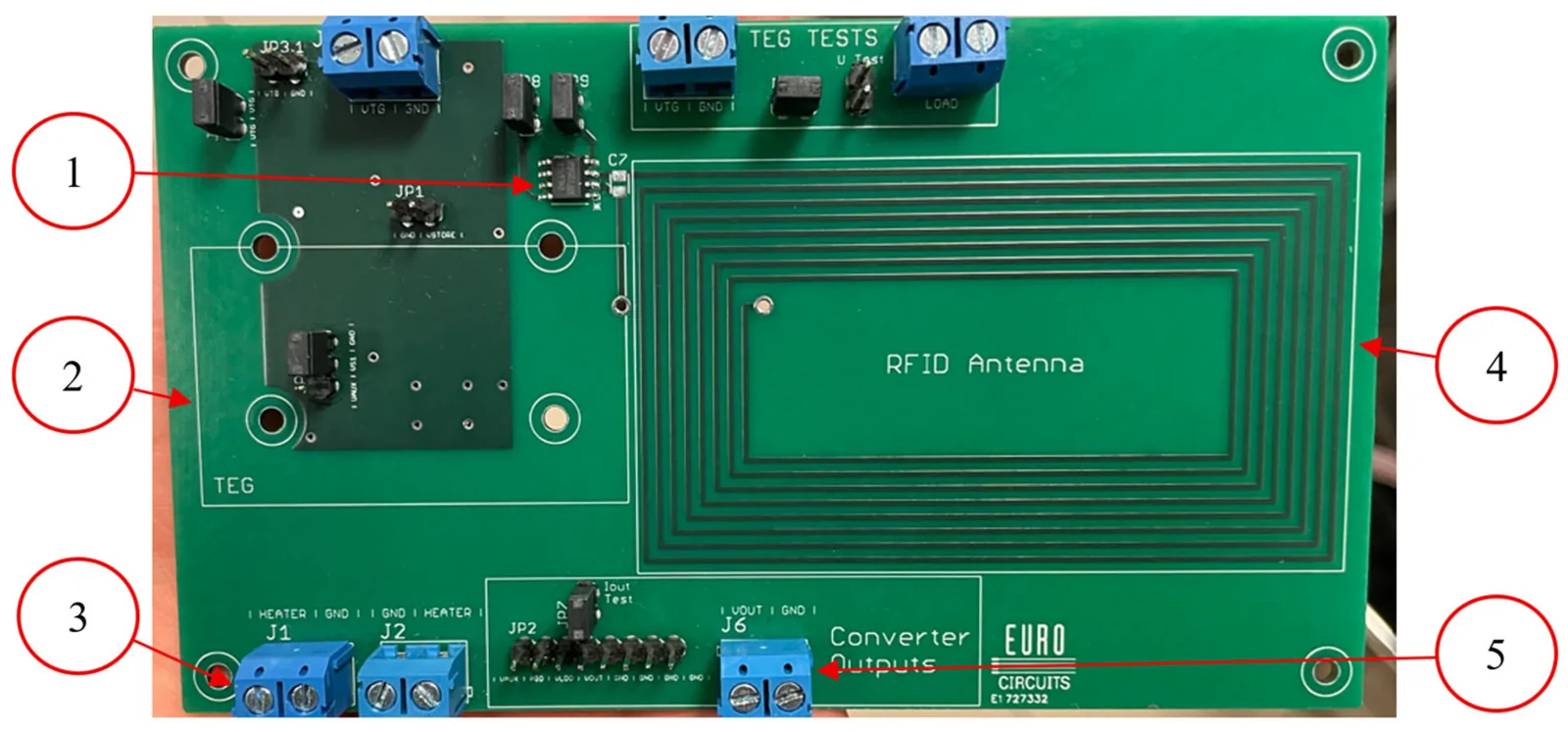
Fabricated PCB—top layer.

**Figure 8:**
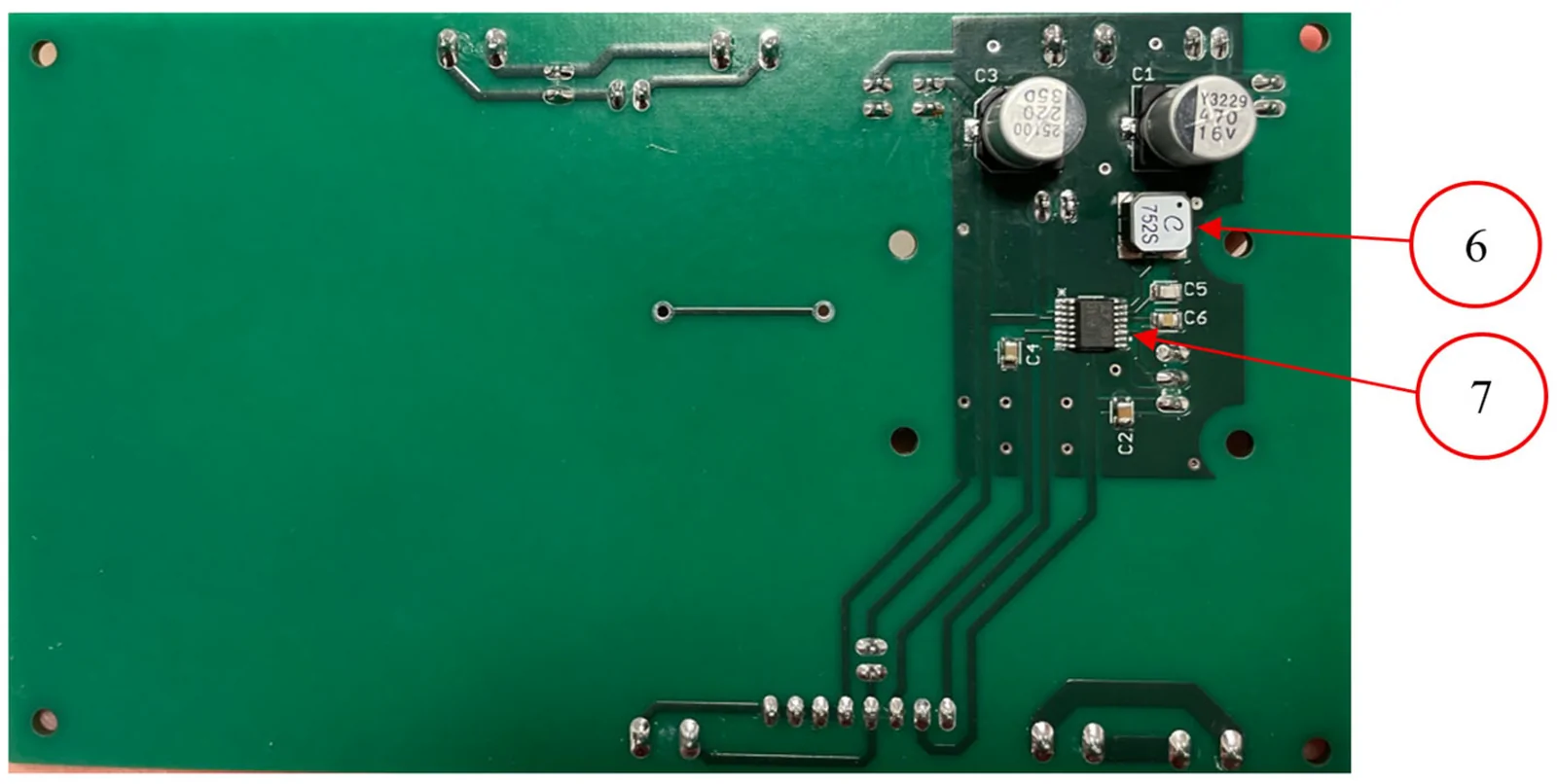
Fabricated PCB—bottom layer.

Based on the developed schematic and the analysis of the connections between the converter and the RFID tag, a printed circuit board was designed, taking into account a range of structural and functional requirements. In particular, provisions were made for mounting the μTEG microgenerator on dedicated supports, enabling efficient heat dissipation from the generator substrate to the surroundings. Special attention was also given to maintaining the proper parameters of the RFID antenna. For this purpose, STM eDesignSuite software (NFC Inductance module) was used to optimize the antenna geometry in order to achieve the required inductance value (5 μH) for the M24LR64E-R device. The developed design meets the electrical and technological requirements necessary for proper system operation. The fabricated PCB is presented in Figure 7 and Figure 8.

In Figure 7 and Figure 8, the following elements can be identified: 1—STM M24LR64E-R RFID tag; 2—μTEG mounting area; 3—connection point for the heater strip power supply; 4—RFID tag antenna; 5—additional output of the converter (by default connected to the RFID tag); 6—1:100 transformer; 7—LTC3108 converter.

**Figure 9:**
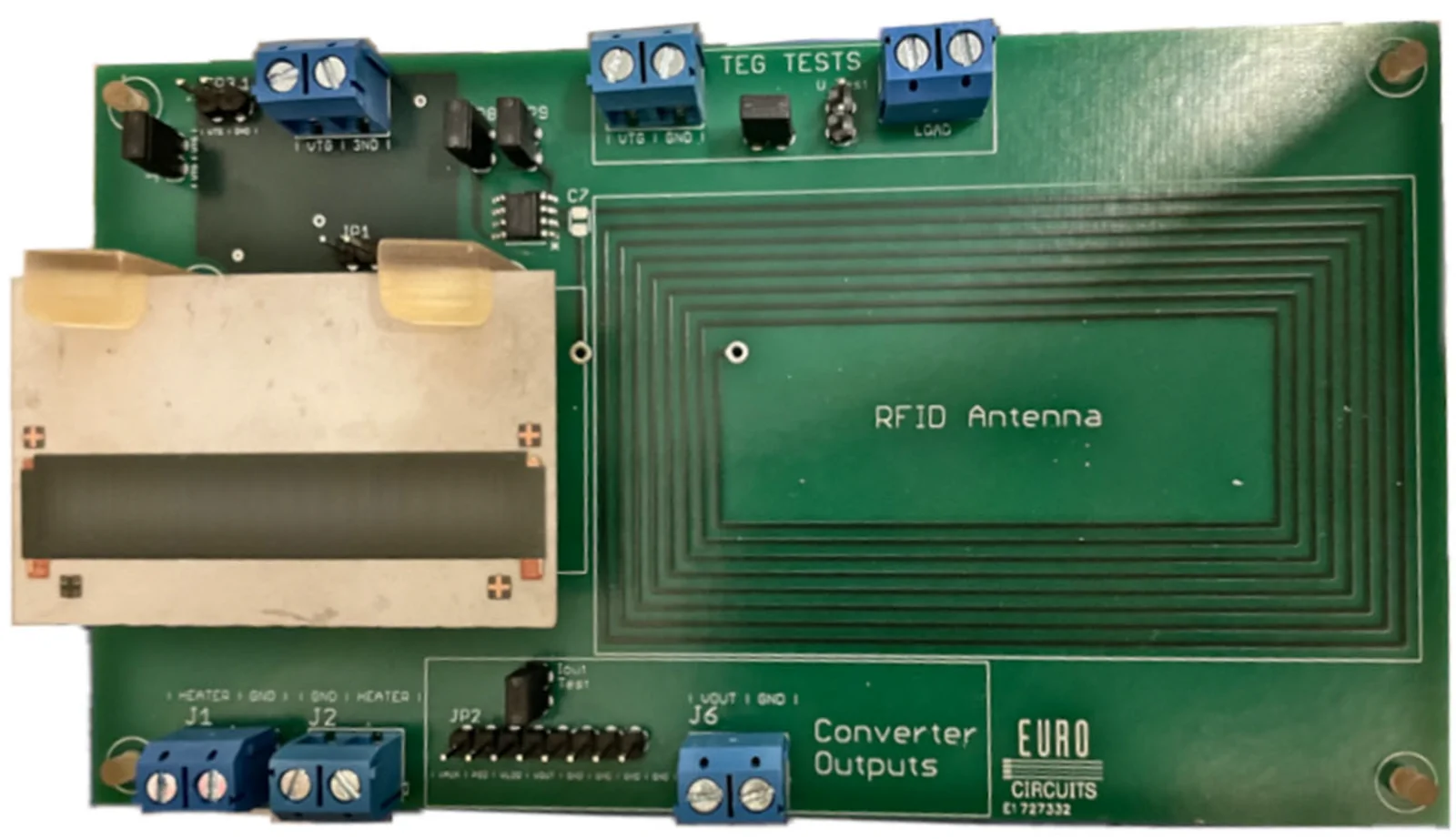
The implemented system integrating the CuNi–Cu μTEG with the STM M24LR64E-R.

The fully integrated board with the mounted CuNi–Cu thermoelectric microgenerator is shown in Figure 9.

## 6. Results

In order to verify the actual power delivered to the converter, the voltage drop at the input of the LTC3108 circuit was measured. In the first stage, the open-circuit voltage of the microgenerator (80 mV) was generated, and then the converter was connected to the circuit. After successful startup, voltage measurements across the load ($U_{LTC}$) were performed. The current in the circuit was determined based on:

(7) $$I=\frac{U_{OC}-U_{LTC}}{R_{TEG}}$$

Subsequently, knowing the voltage drop and the determined current, the input resistance of the converter ($R_{LTC}$) was calculated. Finally, the input power ($P_{in}$) supplied to the system was determined. The obtained results are summarized in Table 3.

As can be observed, the determined converter resistance does not satisfy the $R_{LTC}\approx R_{TEG}$ assumption. Consequently, the system does not operate at the maximum power transfer point. For $U_{OC}$ = 80 mV, under resistive matching between the converter and the microgenerator, the input power $P_{in}$ would be 225 µW.

**Figure 10:**
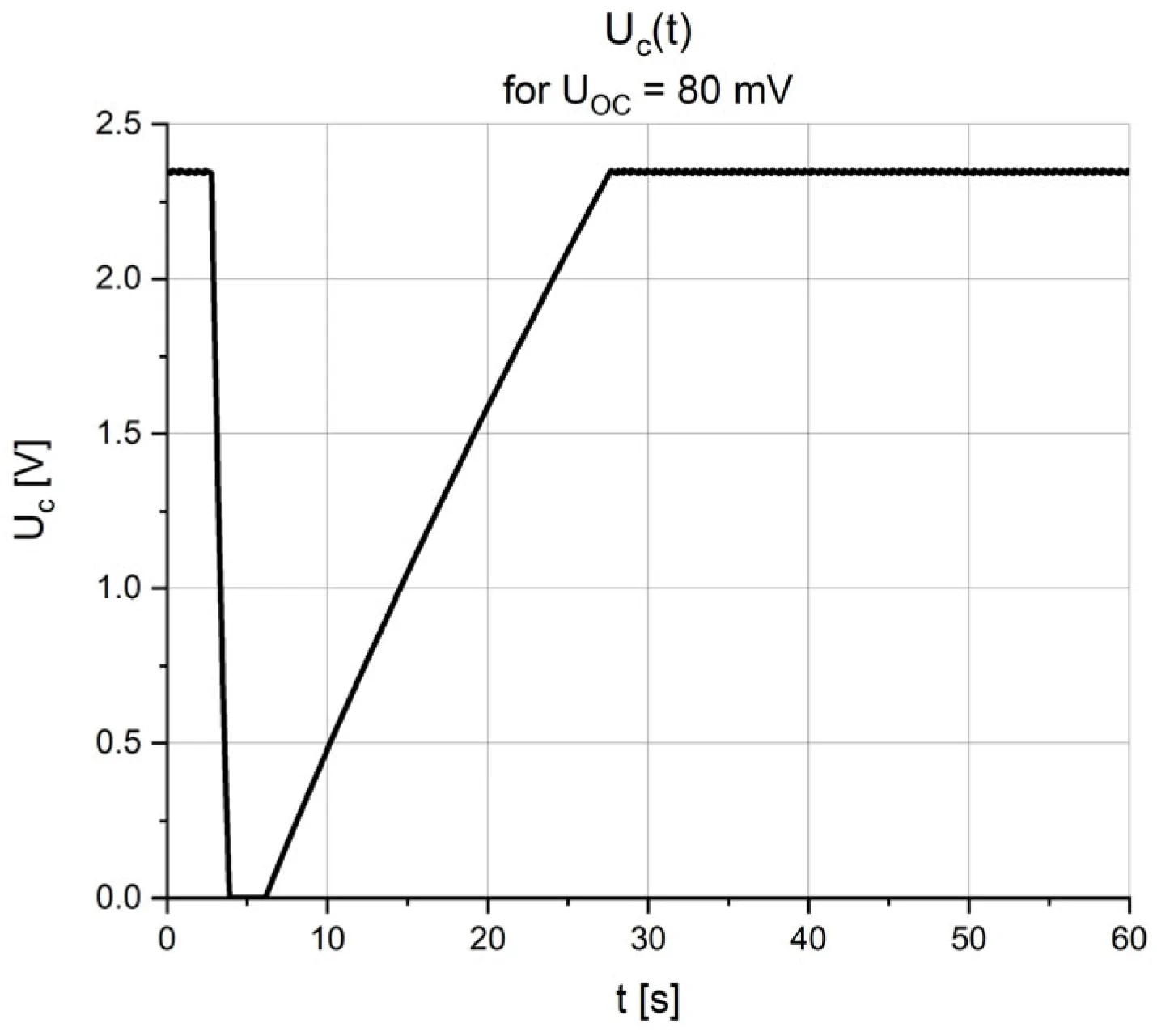
Charging of the output capacitor with the converter supplied at $U_{OC}$ = 80 mV.

Although the LTC3108 system powered only by the μTEG should be able to increase the output voltage to 2.35 V, a 220 µF capacitor was used at the converter output according to the manufacturer’s recommendations. To verify the performance of the voltage boost system, capacitor charging time measurements were performed for the 80 mV open-circuit microgenerator voltage (Figure 10). At the beginning of each measurement, the capacitor was fully discharged, after which the charging process was initiated. As shown in Figure 10, the initial phase of the measurement includes the discharge of the previously charged capacitor, followed by the start of the charging process.

In the system, energy is buffered in an output capacitor with a capacitance of 220 µF, charged to a stable voltage of 2.35 V. The measured full charging of the capacitor under these conditions allows for an estimation of the average power delivered by the converter. The energy stored in the capacitor is given by:

(8) $$E=\frac{1}{2}C\cdot U^{2}$$

where E—energy stored in the capacitor [J]; C—capacitance of the capacitor [F]; U—voltage at the converter output [V].

Knowing the time required to charge the capacitor to this level, the average power delivered by the converter over the considered time interval can be determined. After substitution, the final expression for the average converter power is obtained (Equation (9)).

(9) $$P_{out}=\frac{E}{t_{load}}=\frac{C{\cdot U}^{2}}{2t_{load}}$$

where $t_{load}$—capacitor charging time [s].

Based on the presented relationship, the output power ($P_{out}$) and the efficiency (η) of the converter were subsequently determined using the data summarized in Table 3, in accordance with Equation (10). The obtained results are presented in Table 4.

(10) $$\eta =\frac{P_{out}}{P_{in}}$$

where η—efficiency of the LTC3108 converter system [-].

For $U_{OC}$ = 80 mV, the converter charged the capacitor in 21.66 s, which allows the average output power of the converter to be estimated as $P_{out}$ = 29.3 µW. In practice, this means that in steady-state operation, the average power consumed by the load should not exceed this value if the system is to operate without long-term discharge of the buffer capacitor.

In the final stage of the experiment, the charged system was connected to the RFID tag in order to verify current consumption and observe the discharge dynamics of the buffer capacitor, while maintaining continuous power supply to the converter from the microgenerator with an open-circuit voltage of $U_{OC}$ = 80 mV. The measurement aimed to evaluate the stability of the system under real load conditions and to confirm the system’s ability to maintain power supply.

After charging the capacitor and connecting it to the RFID tag, a dynamic test was performed in which the FEIG CPR74 reader was repeatedly moved closer to and further away from the system, while continuously transmitting the “Inventory” command. Throughout the experiment, the electrical parameters of the system were recorded using a high-precision Keysight N6706C power analyzer, featuring high measurement resolution and the capability to capture dynamic changes. This enabled reconstruction of the dynamic current consumption profile of the tag during active communication.

**Figure 11:**
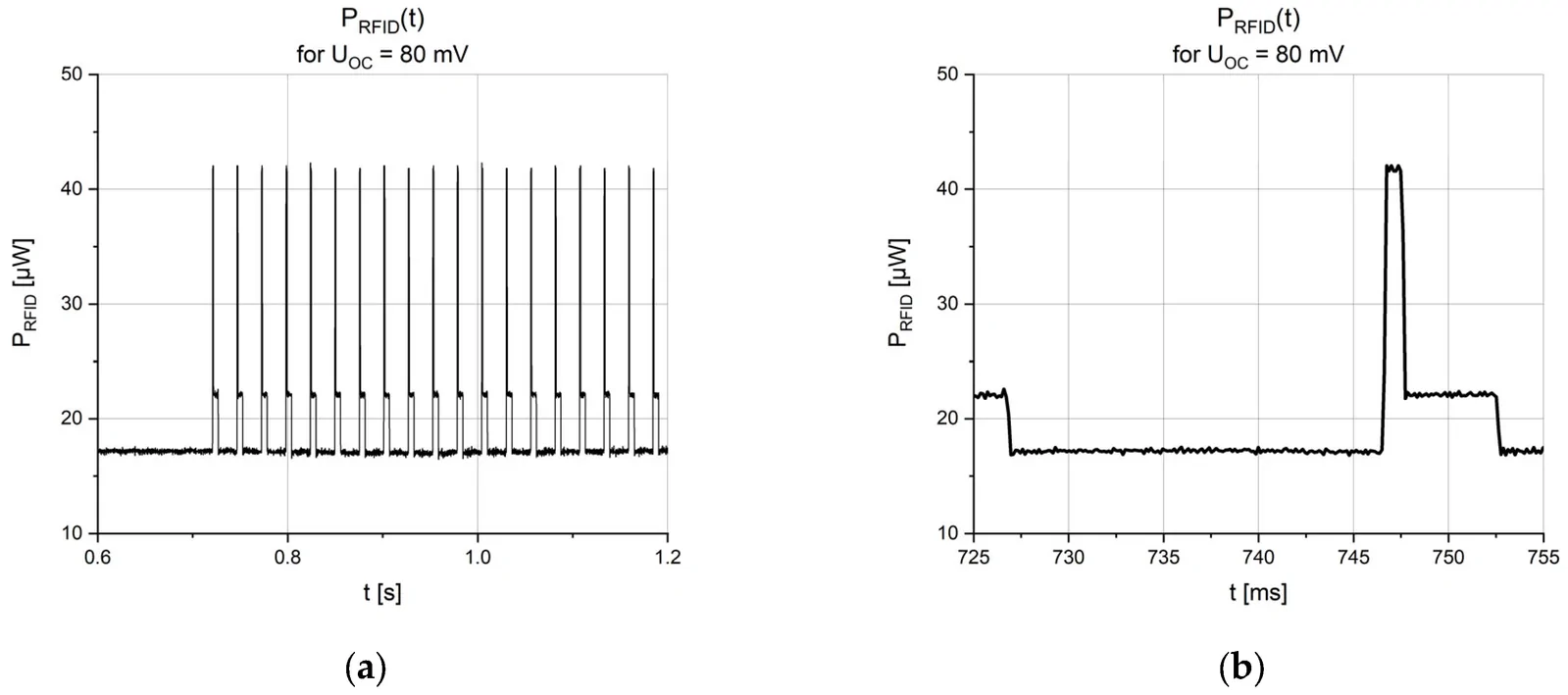
Communication frame during the reader approach to the tag: (**a**) power consumption during the communication cycle; (**b**) power consumption per single bit.

During the acquisition of current waveforms, the RFID tag was continuously powered with a supply voltage of 2.35 V. This allowed the measured current to be converted into the instantaneous power consumed by the RFID tag, as shown in Figure 11.

Due to the correct operation of the system at an open-circuit voltage of $U_{OC}$ = 80 mV, the actual temperature difference between the microgenerator junctions was verified using a FLIR A6703 thermographic camera. The temperature measurements were preceded by calibration of the test setup. The distance between the object and the camera, atmospheric temperature, relative humidity, and apparent (reflected) temperature in the measurement plane were taken into account. To ensure a known, high emissivity value, “Helling 3D Laser Scanning Spray” was applied to the surface under test. As a result, the emissivity of the surface under test was 0.98. Analysis of thermographic images revealed that the temperature distribution within the junction area is non-uniform, which complicates the determination of the actual temperature gradient.

**Figure 12:**
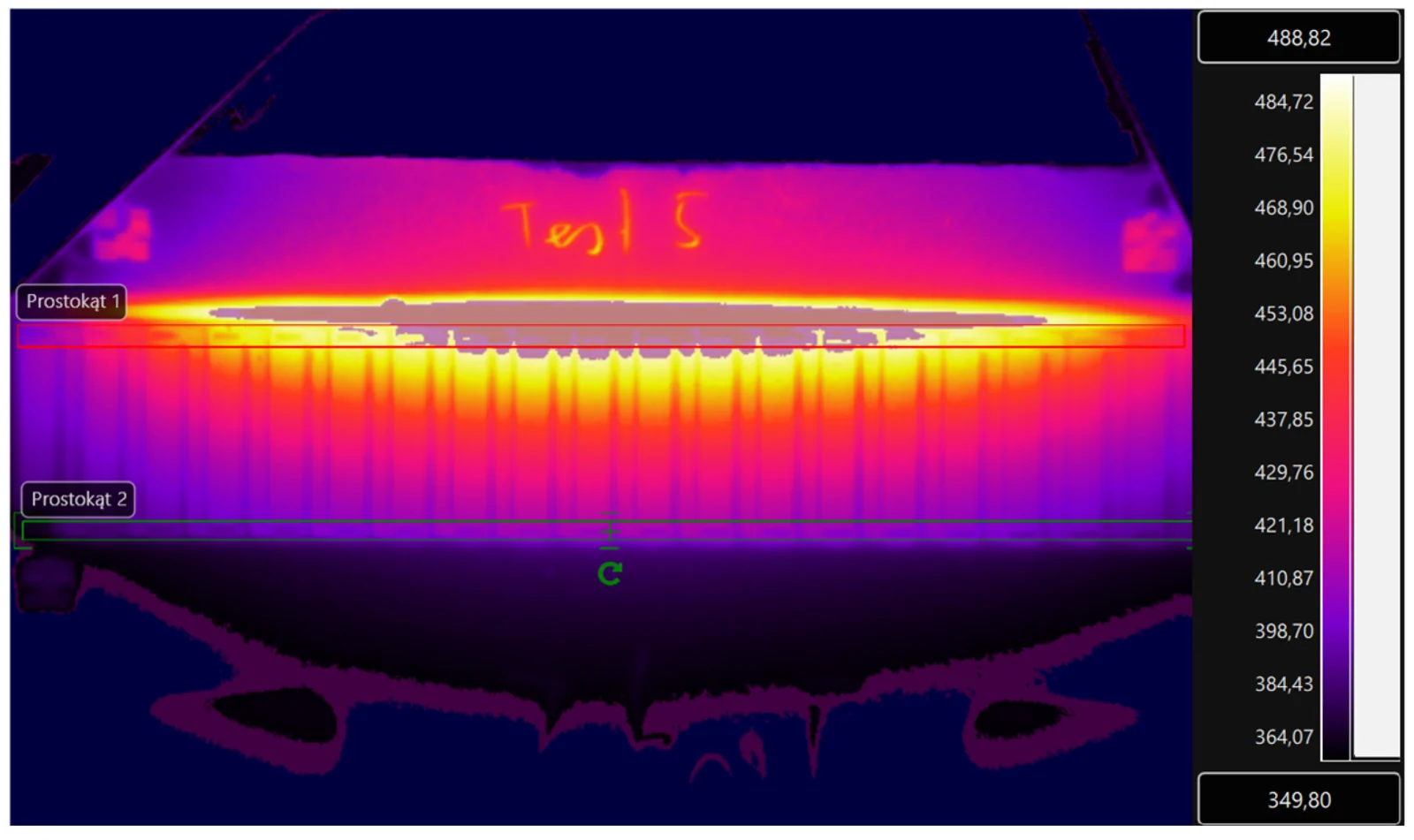
Thermographic image acquired with a FLIR A6703 camera.

To estimate its value, average temperatures were determined for the junction areas. On the thermographic image, rectangular regions of interest covering the analyzed junctions were marked, and the software subsequently calculated the average temperature in each region based on individual pixels (Figure 12).

Thermographic measurements showed that the average temperature on the hot side was 473.16 K, while on the cold side it was 395.09 K. To obtain such a temperature gradient on the alumina substrate between the μTEG hot and cold junctions, the power dissipated on the heating resistor was equal to 11.87 W—the measurements were taken under steady-state conditions. It should be noted that the heating resistor used in this case—made of the same materials as the microgenerator structure (resistive film: CuNi paste; terminations: Cu paste)—was employed solely to confirm the possibility of generating a sufficiently large temperature gradient for a planar CuNi–Cu microgenerator fabricated on an alumina substrate. In practical solutions, such a gradient can be created by connecting one side of the substrate to a waste heat source with a high temperature [20,21]. This automatically generates a large temperature gradient on the substrate, depending on the thermal conductivity of the substrate material and the thermopile arm material, and on the heat convection conditions in the space around the thermoelectric structure (free or forced convection). The simulations presented in [21] show that on substrates with lower thermal conductivity, temperature changes are much greater near the hot junctions of the thermopile, i.e., a larger temperature gradient appears at this distance from the hot ends of the thermopile. This phenomenon is further amplified if forced convection occurs near the microgenerator.

## 7. Discussion and Conclusions

Based on the communication waveform, it can be concluded that in the idle state the tag consumes approximately 17 µW of power. During data exchange, instantaneous power consumption increases to approximately 42 µW for 1 ms and then remains at 22 µW for the subsequent 5 ms. After this cycle, the power consumption drops again to approximately 17 µW and remains at this level for about 20 ms until the next peak occurs. Based on the experimental data, the average power consumption of the tag over the entire communication cycle was determined to be 19.06 µW. The absence of any observed voltage drops on the capacitor, which maintained a stable voltage of 2.35 V, indicates a positive energy balance of the system. This means that the power supplied by the converter is sufficient to cover the demand of the tag, and the short-term increases in power consumption are compensated during periods of lower load, when the consumed power falls below $P_{out}$ = 29.3 µW.

The value of $P_{out}$ is exceeded only for approximately 1 ms, while for the remaining 25 ms of the cycle the power consumption remains significantly lower than the converter output power. On this basis, it can be concluded that the system properly supplies the RFID tag, thereby contributing to the improvement of its operating performance. It is worth noting that the average power consumption of 19.06 µW is also significantly lower than the available average power $P_{out}$.

In summary, the planar μTEG microgenerator can be successfully used to power the STM M24LR64E-R RFID tag with the aid of an LTC3108 converter. The analysis of the output power balance indicates that the available power is sufficient to ensure stable operation of the system. Consequently, this may lead to an improvement in the RFID tag performance, such as an increased communication range and a reduced response time during transmission initiation. This results from the fact that the internal circuitry of the tag is continuously powered, eliminating the need for repeated wake-up procedures prior to communication.

Based on the conducted thermographic measurements, it was found that the average temperature on the hot side was 473.16 K, while on the cold side it was 395.09 K. This corresponds to a temperature difference between the junctions of approximately 78 K. Assuming an open-circuit voltage of 80 mV, this gives a theoretical Seebeck coefficient of a single CuNi–Cu thermocouple of 51.28 µV/K. It should be emphasized that this value was determined based on averaged temperatures within the measurement regions (between 395 K and 473 K). According to previous analyses and measurements, the expected value of the global Seebeck coefficient was approximately 42.86 µV/K. However, the measurements of the local Seebeck coefficient, presented in [17], show that in this range it reaches values above 50 µV/K, demonstrating consistency with thermographic measurements.

Considering the obtained results, a temperature difference of 68 K would allow an open-circuit voltage of 70 mV to be obtained, resulting in an output power of the LTC3108 converter of $P_{out}$ = 20.27 µW. This value still satisfies the average power requirement of the tag over the entire communication cycle, which is 19.06 µW, and indicates that the system could theoretically operate at a lower temperature gradient.

Finally, the parameters obtained from simulations were compared with those measured experimentally. This comparison made it possible to assess the extent to which the simulations reflected the actual behavior of the system. The data are summarized in Table 5.

The simulation results obtained allowed full integration of the planar thermoelectric microgenerator made of CuNi–Cu materials, with an internal resistance of 7.11 Ω and consisting of 20 thermocouples. However, it should be noted that the actual input resistance of the LTC3108 converter does not match the resistance of the microgenerator, which constitutes the main source of discrepancies between the simulation and experimental results.

Despite these limitations, acceptable agreement was obtained between the values determined in simulations and those measured experimentally. Although a slightly higher converter efficiency was expected based on the literature data, the achieved output power proved sufficient to properly power the RFID tag, which was the main objective of the conducted analysis.

The conducted study confirms the significant potential of thermoelectric structures in energy harvesting applications. The investigated use case for powering RFID tags represents only one of many possible applications of TEG generators. In the case of the CuNi–Cu structure, a relatively large temperature difference between the junctions was required; however, such conditions may naturally occur in various industrial and environmental processes.

The proposed μTEG–RFID system is not intended to replace conventional passive RFID tags, but rather to address applications in which a continuous source of waste heat is naturally available, enabling long-term autonomous operation without the need for a battery. Such conditions can be found in industrial installations and transportation systems, where elevated temperatures are present during normal operation, for example, in steam and hot-water pipelines, heat exchangers, industrial furnaces, electric motors, pumps, and other process machinery. Promising application areas can also be found in the automotive sector, where exhaust manifolds, exhaust pipes, catalytic converters, turbochargers, and engine blocks constitute permanent sources of waste heat. In these environments, the μTEG can continuously provide energy to the RFID system, allowing the tag to remain in an active state and respond immediately to interrogation without requiring a wake-up sequence from the RF field.

The availability of thermal energy sources in the operating environment and their potential utilization for thermoelectric energy harvesting have been extensively investigated. In the present study, the external power supply was used solely to establish controlled and repeatable thermal conditions for experimental characterization of the proposed μTEG–RFID system and does not represent the intended heat source in practical applications. Instead, the required temperature gradient is expected to be provided by naturally available waste heat generated during the normal operation of heat-generating systems. Numerous studies have demonstrated that such systems provide reliable sources of high-temperature waste heat, with operating temperatures exceeding 473 K, which can be effectively converted into electrical energy using thermoelectric generators. The feasibility of such an approach has been confirmed in various thermoelectric waste heat recovery applications. Experimental investigations of thermoelectric generators applied for vehicle exhaust heat recovery have demonstrated operation with hot-side temperatures of approximately 473 K, confirming that thermal conditions comparable to those used in the present study can be achieved from naturally available waste heat sources [22]. Similarly, thermoelectric generators integrated with diesel engine exhaust waste heat recovery systems have been reported to operate with hot-side heat exchanger temperatures ranging from 458 to 476 K, depending on the operating conditions [23].

Furthermore, industrial waste heat recovery studies have demonstrated the possibility of utilizing sources with even higher temperature levels, including process heat streams exceeding 500 K [24]. A comprehensive review of thermoelectric waste heat recovery technologies has also highlighted the suitability of thermoelectric generators for converting otherwise dissipated exhaust and process heat into distributed electrical power, particularly in applications requiring autonomous operation and continuous energy availability [25]. Overall, the reported results confirm that the temperature gradient applied in this work represents a realistic operating condition for selected waste heat harvesting scenarios. In practical implementations, the required thermal input would be provided by heat losses generated during normal operation of industrial or automotive systems, enabling long-term autonomous operation of the proposed μTEG–RFID platform.

It should be noted, however, that the temperature difference of approximately 78 K demonstrated in this study refers only to the local temperature gradient across the thermoelectric substrate between the hot and cold junctions of the μTEG and is not expected to occur across the entire RFID system. In practical implementations, such a gradient can be achieved by thermally coupling one side of the μTEG to a heat source while providing an effective heat dissipation path on the opposite side. Moreover, the required temperature difference can be further reduced by optimizing the μTEG geometry, including the number and dimensions of thermocouples, thermoelectric material thickness, and thermal design of the substrate, which may enable operation under less demanding thermal conditions. The presented structure was designed as a proof of concept optimized for the selected LTC3108 converter and available fabrication constraints; therefore, further optimization of the microgenerator design may improve its suitability for applications with lower temperature gradients.

It is also important to emphasize that the μTEG and the RFID tag do not necessarily need to be located in close proximity. Depending on the specific application, the microgenerator can be placed directly at the heat source, while the RFID electronics and antenna can be positioned at a thermally safer location and connected through appropriate electrical connections. Such spatial separation enables better thermal management and allows the RFID components, including the integrated circuit, PCB, antenna structure, solder joints, and polymer-based elements, to operate within their allowable temperature ranges. Therefore, the proposed concept is not intended for direct attachment to arbitrary high-temperature surfaces. It should be noted that the presented results were obtained under controlled laboratory conditions as part of a proof-of-concept experimental study and should not be interpreted as representing the final implementation in a practical application.

Thermoelectric microgenerators represent a promising solution for harvesting waste energy, and further development of this technology may contribute to improved energy efficiency and reduced energy losses, which in the long term may have a beneficial impact on the environment.

## Figures and Tables

**Table 1:** Summary of the materials used.

Material	Supplier	Catalogue Number	Drying Temperature [K]	Maximum Firing Temperature [K]	Firing Time [min]
Cu	Fraunhofer IKTS	FK3101 E02	393	1123	60
CuNi	Fraunhofer IKTS	W22016 E01	393	1123	60
Glaze	Heraeus	IP7098	423	1003	20

**Table 3:** Results obtained at the input of the converter.

U_OC_ [mV]	U_LTC_ [mV]	I [mA]	R_LTC_ [Ω]	P_in_ [µW]
80	32.4	6.69	4.84	216.76

**Table 4:** Results obtained at the output of the converter.

U_OC_ [mV]	t_load_ [s]	P_out_ [µW]	η [%]
80	21.66	29.25	13.52

**Table 5:** Summary of the obtained values.

U_OC_ [mV]	U_LTC_ [mV]		R_LTC_ [Ω]		P_in_ [µW]		P_out_ [µW]		η [%]	
	Sim.	Meas.	Sim.	Meas.	Sim.	Meas.	Sim.	Meas.	Sim.	Meas.
80	40	32.4	7.11	4.84	225.04	216.76	33.76	29.25	15	13.52

## Data Availability

Data are contained within the article.
